# Soft-Tissue Volume Augmentation at Dental Implant Placement Using Collagen-Based Matrix Characterized by Oriented Open Pore Structure: A Retrospective Study with a Median Follow-Up of 17 Months

**DOI:** 10.3390/bioengineering12121324

**Published:** 2025-12-04

**Authors:** Bastian Wessing, Bouke Boekema

**Affiliations:** 1 Zahnkultur Marzahn Medical Care Center, 12681 Berlin, Germany; 2 Dental Practice Clinic, Luisenhospital, 52064 Aachen, Germany; 3 Preclinical Research, Association of Dutch Burn Centres, 1940 AE Beverwijk, The Netherlands; bboekema@burns.nl

**Keywords:** biocompatibility, collagen matrix, dental implant, esthetics, histology single implant, soft tissue augmentation

## Abstract

**Background/Objective:** Soft-tissue volume augmentation with gingival grafts enhances implant-prosthodontic esthetics by maintaining or reconstructing a convex contour of the vestibular mucosa. However, it presents disadvantages related to donor site, surgical complexity, and post-procedural discomfort. This study evaluated clinical performance of a native porcine-derived collagen–elastin matrix with uniquely oriented porous structure for soft-tissue volume augmentation at single implant sites. **Method and Materials:** Soft-tissue augmentation was performed at sites of a single implant, which underwent immediate, early, or late loading protocols. Implant success, its survival, and mucosal health status including change in tissue thickness acquired through 3D scans of casts prepared from impressions before and after tissue augmentation were evaluated. Biocompatibility of the collagen matrix (CM) was assessed through histological analyses. **Results:** Forty-five patients received 50 implants with simultaneous augmentation using CM. At the last follow-up (mean 22.1 ± 15.0 months), the peri-implant soft tissue was healthy and stable, and all implants were surviving and successful. Tissue thickness change at augmented sites did not vary with time interval between pre- and post-treatment, indicating stability of augmentation. Soft-tissue biopsies (n = 3) showed healthy peri-implant soft tissue with good vascularization and no inflammation. **Conclusions**: This retrospective analysis demonstrated good clinical performance and high biocompatibility of CM for soft-tissue volume augmentation around dental implants.

## 1. Introduction

Following tooth extraction, the alveolar ridge undergoes a physiological resorption, mainly at the buccal site. It has been shown that resorption of approximately 50% of the alveolar crest width occurs in the first 12 months after tooth extraction; two-thirds of this resorption occur within the first 3 months [1]. Numerous studies have shown that it is possible to regenerate alveolar bone either with autologous bone or bone substitute materials, both demonstrating comparable results [2,3].

Soft-tissue volume augmentation with gingival grafts at dental implant sites are known to enhance implant-prosthodontic esthetics by maintaining or reconstructing a convex contour of the vestibular mucosa and/or by preventing the titanium abutment from shining through a thin buccal bone plate and mucosa [4,5]. Furthermore, thick peri-implant mucosa prevent soft-tissue recession [6], whereas a thin, soft tissue, with less than 2 mm thickness, led to increased bone resorption during the first year after the re-entry procedure [7].

Techniques utilizing autologous subepithelial connective tissue and de-epithelialized or free gingival grafts from the palate [8], as well as tuberosity grafts, are commonly used to prevent esthetic failures and peri-implant disease [9,10] and are considered to be the “gold standard”. However, depending on the donor site (anterior palate, posterior palate, or tuberosity area) or the technique, the quantity and quality of the available material may vary [11,12]. Although a randomized clinical trial evinced a greater mean root coverage following the connective tissue graft (CTG) compared to xenogeneic collagen matrix (XCM) [13], several studies demonstrated the drawbacks of using CTG, including the need for a second surgical site and problems such as a complicated surgical procedure, post-surgical bleeding, edema, and pain, in addition to the limited volume of autologous tissue [14,15,16,17]. These conclusions are supported by recent systematic reviews comparing XCM vs. free gingival graft [18] or subepithelial CTG [19], which found more favorable outcomes for autologous grafts in terms of the resulting thickness. However, the considerably shorter surgical time, lower post-operative pain, and the overall no significant difference in esthetics (despite various assessment methods) were reported when XCM was applied. Authors suggested that these factors, along with treatment goals, should be considered when selecting a grafting approach, acknowledging that evidence from their analysis carries moderate certainty and necessitates further studies. Therefore, a search continues [20] for suitable soft tissue substitutes with high biocompatibility and unlimited quantity, which show consistent results and no further co-morbidities. The efficacy of such substitute materials for soft-tissue regeneration is still being investigated.

One of such soft-tissue substitute materials is creos^TM^ mucogain (CM), a porcine-derived native collagen-elastin-based three-dimensional heterogeneous matrix with a modified interconnecting pore structure. Its uniquely oriented porous structure has been shown in vitro [21] to promote active migration of collagen-producing human dermal fibroblasts and in vivo to facilitate soft-tissue regeneration by guiding migrating cells and blood vessels, as observed in a porcine implantation study [22]. The matrix is not chemically cross-linked and therefore is less likely to evoke an immune response, as suggested by in vivo animal models [23,24]. In patients, it was successfully applied to treat gingival recessions [25].

This retrospective study offers certain advantages by including real-world variability. Unlike controlled trials with rigid protocols, retrospective real-world studies capture the diversity of patients, procedures, and clinical conditions encountered in daily practice [26]. The aim of this investigation was to evaluate the clinical performance of the CM for soft-tissue volume augmentation at single implant sites.

## 2. Materials and Methods

### 2.1. Ethical Considerations

The ethics committee of the medical association of North Rhine-Westphalia, Germany, formally granted exemption approval due to the design of the study in accordance with German legislation (§15; 203/2018). The study was registered in the German Clinical Trials Register (DRKS) and the International Clinical Trials Registry Platform (www.who.int; DRKS00015213).

### 2.2. Study Design and Participants

A retrospective single-center study was conducted to assess the clinical efficacy of CM (creos mucogain; Nobel Biocare AB, Gothenburg, Sweden; previously distributed as Mucomaix, Matricel GmbH, Herzogenrath, Germany) for soft-tissue volume augmentation. The study included consecutive patients who received single implants (NobelActive, NobelParallel CC, NobelReplace CC, NobelReplace Straight Groovy, or NobelPearl; Nobel Biocare AB) simultaneously with soft-tissue volume augmentation using CM, between 28 September 2013 and 25 May 2018, at a German private dental practice. Soft tissue augmentation was performed in cases were the gingival thickness was <2 mm to avoid biological complications [7] and/or when vestibular soft tissue contour deficiencies were present for esthetic reasons [27]. General exclusion criteria were defined as (1) being <18 years of age, (2) undergoing treatment with an interfering medication, such as steroids or bisphosphonates ≤3 months prior to implant placement surgery and/or during the follow-up, (3) heavy smoking (>10 cigarettes/day), and having the following: (4) acute, untreated periodontitis; (5) any disorders in the planned implant area, such as previous tumors, chronic bone disease, or previous irradiation; (6) a history of past or ongoing alcohol or substance abuse; and (7) uncontrolled diabetes during the surgery and/or follow-up period. Implants underwent immediate, delayed, or late loading protocols in a flapless or flap procedure. Soft-tissue augmentation (STA) was performed using the “envelope technique” [28], a trapezoid-shaped combination of mucosal and mucoperiosteal flaps, or with a coronally advanced combination of mucosal and mucoperiosteal flap, where the sites underwent closed or transmucosal healing. The matrix was rehydrated using sterile saline or blood from the operation sites. Bone grafting was performed using guided bone regeneration (GBR) using the “Sandwich technique” according to Hom-Lay Wang [29], a gap augmentation (coupled with immediate loading protocol), or in a sinus elevation procedure. The bone graft materials comprised autologous bone chips with NanoBone (Artoss GmbH, Rostock, Germany), Bio-Oss (Geistlich Biomaterials, Wolhusen, Switzerland), or creos xenogain (Nobel Biocare AB). Provisional prostheses were screw- or cement-retained. The final prostheses consisted of either cement-retained porcelain-fused-to-metal (PFM) crown with a Titanium Procera abutment, Zirconia crown with a pre-fabricated Zirconia abutment, Zirconia crown with a Procera Titanium abutment, Zirconia crown with a Procera Zirconia abutment, Zirconia crown with a Zirconia abutment, or the screw-retained veneered angulated screw channel abutment and veneered Procera Esthetic abutment (Nobel Biocare AB). STA-related complications assessed at 1 and 3 weeks after the surgery included matrix exposure, residual swelling and pain, and wound dehiscence with matrix exposure. All treatment procedures and relevant clinical data in the practice (including patient-reported pain assessment rated on a scale of 1−10, where 1 = low discomfort, 10 = high discomfort) were routinely recorded in the electronic health record (Charly; Solutio GmbH, Holzgerlingen, Germany).

### 2.3. Data Extraction

Data extraction was performed from the above-mentioned electronic health record system, Charly, which stored routinely obtained patient surveys and clinical data registration. Extracted data included patient information such as age, gender, general medical history (diabetes, infectious diseases, etc.), medication intake, periodontal health status including bleeding on probing (BOP), approximal plaque index (API) measured according to Lange [30], pocket depth, papilla index scored according to Jemt [31] (0, no papilla was present; 1, less than half of the papilla height was present, and a convex curvature of the soft tissue contour adjacent to the implant crown and the adjacent tooth was observed; 2, half or more of the papilla height was present, but not extending to the contact point between the teeth; 3, the papillae filled the entire proximal space, were harmonious with the adjacent papillae, and presented an optimal soft tissue contour; or 4, the papillae were hyperplastic and covered too much of the implant restoration or adjacent tooth, with an irregular soft tissue contour), keratinized soft-tissue height, along with reason for tooth loss, implant parameters, use of biomaterials, surgical procedures, post-operative complications (dehiscence, matrix exposure, or inflammation), and prosthetic solutions. Implant survival and success [32] were also recorded.

### 2.4. Augmentation Analysis

Polyether impressions (Impregum Penta, 3M ESPE, Neuss, Germany) were taken routinely before implant placement with simultaneous STA for drill splint manufacturing, and at the time of impression-taking for the final restoration. Dental casts were fabricated from each impression by an experienced laboratory technician and subsequently stored. However, at the time of analysis (in certain cases, more than 4, 5 years after treatment), some were missing or have sustained damage rendering them unreadable, leaving 26 casts. Of these, four were from patients who received simultaneous lateral bone augmentation. Due to the limited number of readable casts, all available ones were scanned using a 3D laser scanner (L2i, Imetric, Courgenay, Switzerland).

**Figure 1:**
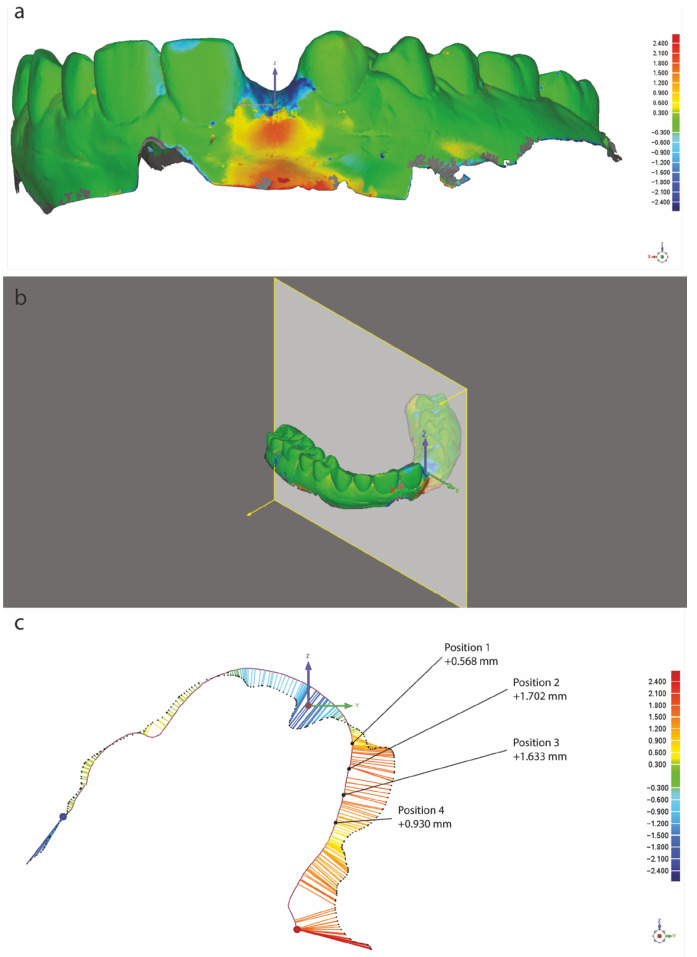
Example of quantitative three-dimensional methodology to assess the outcome of volume augmentation. (**a**) Digital model after superimposition of the STL files collected at pre-treatment (T0) and impression-taking for the final prosthesis (T1). (**b**) Selection of the sagittal section for the 2D comparison. (**c**) Two-dimensional comparison of the superimposed models on the middle of the augmented area. The distance between the pre-operative (solid line) and post-operative (dashed line) soft-tissue profile is color-coded, with green indicating minimal changes, red indicating soft-tissue gain, and blue indicating soft-tissue loss.

The STL files obtained from each model were subsequently transferred to a digital shape sampling and processing software (Geomagic Qualify 12, 3D Systems, Rock Hill, SC, USA) for re-elaboration of the 3D models obtained from the 3D scan data. For each patient, pre-surgical (T0) and post-surgical (T1) models were superimposed, based on a procedure that relies on the best matching of manually selected surfaces. The Geomagic software was then used to perform an automatic alignment and superimposition of the two best matched models into one coordinate system. A superimposition between the best matched models was achieved using 300 randomly selected points to obtain the initial orientation. Further fine adjustments based on 1500 points were made to achieve the final alignment. Prior to measurements, the pre-surgical model was set as reference, and the post-surgical model was set as test. To assess the augmentation outcome for each patient, an area of interest at the vestibular aspect of the edentulous gap was defined, and the change in alveolar ridge thickness in this area was evaluated. For each superimposition, sagittal sections perpendicular to the axis of the healing abutment were obtained and one sagittal section corresponding to the mesio-distal center of the abutment was selected. The distance between the pre-operative and post-operative mucosal profiles was measured at four locations for each profile, 1 mm apart, in an apical direction from the mucosal margin (Figure 1) [33].

### 2.5. Immunohistochemical Analysis

After submerged healing, if abundant keratinized mucosa was present, a punch technique approach was utilized during the re-entry procedure to place the healing abutment. This provided material for biopsies without additional burden on patients (thus not requiring Ethical Committee approval). The punch technique was applied exclusively in the cases with sufficiently thick keratinized mucosa with an adequate soft tissue contour, thus resulting in a limited sample size (5 samples from 3 patients). Although the procedure is not considered standard of care, it was performed only when risks, such as the loss of necessary keratinized tissue, were not posed, with patient’s consent being obtained. Tissue samples (punch biopsy, Ø 3 mm) were fixed in formalin and processed for paraffin embedding. Sections (5 µm thick) were de-paraffinized, rehydrated, and subsequently stained using standard techniques. Standard hematoxylin and eosin and Herovici [34,35] staining (Supplementary Material) were used to visualize general organization of the tissues. Blood vessels were visualized with primary antibodies against von Willebrand factor (polyclonal, cat F3520 Sigma-Aldrich, St. Louis, MO, USA; 1:5000 dilution; Supplementary Material), CD34 (clone QBend-10, cat M7165 Dako, Glostrup, Denmark; 1:300 dilution; Supplementary Material), collagen IV (clone CIV 22, cat M0785 Dako; 1:100 dilution; Supplementary Material), and α-smooth muscle actin (α-SMA; clone 1A4, cat M0851 Dako; 1:500 dilution), macrophages with anti-CD68 antibodies (clone KP1, cat M0814 Dako; 1:200 dilution), and CM remnants with anti-elastin antibodies (clone BA-4, cat E4013 Sigma-Aldrich; 1:500 dilution). Brightvision poly-horseradish peroxidase anti-mouse IgG and Bright DAB Solution (Immunologic, Duiven, The Netherlands) were used for visualization of primary antibodies. Images were captured at 50× and 200× magnification (NIS Elements Ar software v 4.60, Nikon, Amsterdam, The Netherlands).

### 2.6. Statistical Methods

Descriptive statistics, including frequency tables, were used for the presentation of results as well as boxplots and scatterplots. Statistical analyses were performed at patient and implant levels, but for STA, analysis was performed at the measuring point level. All descriptive statistical and graphical analyses were performed using SPSS Statistics 24 (IBM, Armonk, NY, USA). Pearson’s and Spearman’s rank correlation coefficient tests were used to assess the possible impact of time (T0 to T1) on changes in tissue thickness. The Kruskal–Wallis rank sum test was used to compare changes in tissue thickness at extraction vs. recently healed vs. healed sites and the Mann–Whitney U test for pairwise comparison between these groups. The variance within each group was tested using the variance test. All statistical tests were performed in R for Windows version 3.5.1 [36]. Distribution of Jemt’s papilla score was tested using a contingency table.

### 2.7. RECORD Compliance Statement

The Reporting of studies Conducted using Observational Routinely collected health Data (RECORD) guidelines were used as the framework for this study and report [37].

## 3. Results

### 3.1. Patient Demographics, Surgical Procedure and Follow-Up

The study included a total of 45 consecutive patients, of whom 25 were female (55.6%). The population was predominately young (41.4, range 20–66 years) and healthy. The baseline demographic characteristics as well as surgical details are listed in Table 1. All 50 implant sites (three patients were treated with 2 and one patient with 3 implants) received STA using CM (3 mm × 15 mm × 20 mm), trimmed to fit the defect. Provisional restorations were placed on 18 implants (36%); of these, 11 (22%) were immediate, and 7 (14%) were delayed. The majority (64%) of implants did not receive a provisional prosthesis. The provisional prostheses that were placed were mostly screw-retained (n = 15); only three were cement-retained. All implants received a final abutment and restoration. Final restorations were either cement-retained (44%, n = 22 implants) or screw-retained (56%, n = 28), and included veneered angulated screw channel abutments (n = 23), PFM crowns with Titanium Procera abutments (n = 12), veneered Procera Esthetic abutments (n = 5), and Zirconia crowns with Zirconia abutments (n = 5), with Procera Zirconia abutments (n = 2), with Procera Titanium abutments (n = 2), or with a pre-fabricated Zirconia abutment (n = 1). The final prosthesis delivery took place, on average, 5.0 ± 2.5 months (range: 1.5–12.7 months) after implant placement. The last follow-up visit was, on average, 22.1 ± 15.2 months (range: 2.8–53.8 months) after implant placement.

### 3.2. Clinical Outcomes

**Figure 2:**
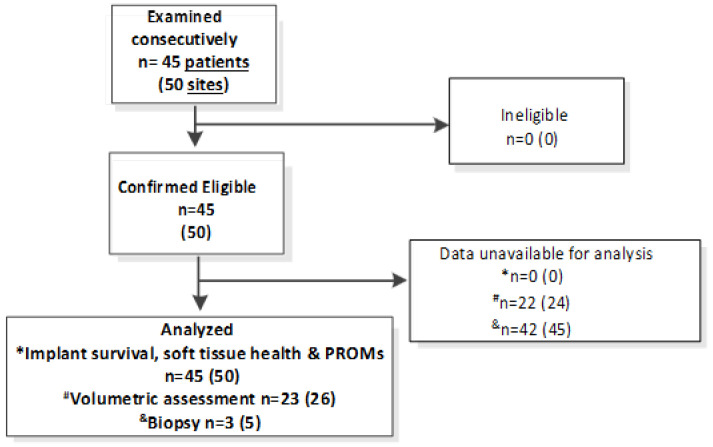
Study flowchart diagram. Due to the retrospective design, the data available for analysis was limited to the routinely collected records. Volumetric data of 19 patients and biopsy data of 42 patients were not available and thus not included in the analysis.

Eligibility criteria for this retrospective study were the same as the general criteria required to be eligible for implant treatment at the practice where the study was conducted. Thus, all the patients who were accepted for dental treatment were automatically eligible. Patient eligibility and data availability is summarized in the study flowchart diagram (Figure 2).

#### 3.2.1. Post-Surgical Complications and Patient Discomfort

Post-surgical complications were assessed at 1 and 3 weeks after implant placement. The complication rate was low, at 1 week being 8% (n = 4), with one central exposure of the matrix, one wound dehiscence with matrix exposure, and two sites with residual swelling and pain. At 3 weeks, the complication rate decreased to 4% (n = 2), with one central exposure of the matrix and one wound dehiscence with matrix exposure (same sites as at 1 week post-surgery). Both exposures closed within the following week without further treatment procedures. A weekly follow-up schedule was carried out in these cases. None of the implants showed signs of infection at 1 or 3 weeks post-surgery. A minimal post-surgical pain score of 1 was reported by patients at 66% (n = 33) of treatment sites, while the remaining 34% of sites had pain scores ranging from 2 to 5, with no sites experiencing score ≥ 6.

#### 3.2.2. Soft-Tissue Health and Implant Survival and Success

**Figure 3:**
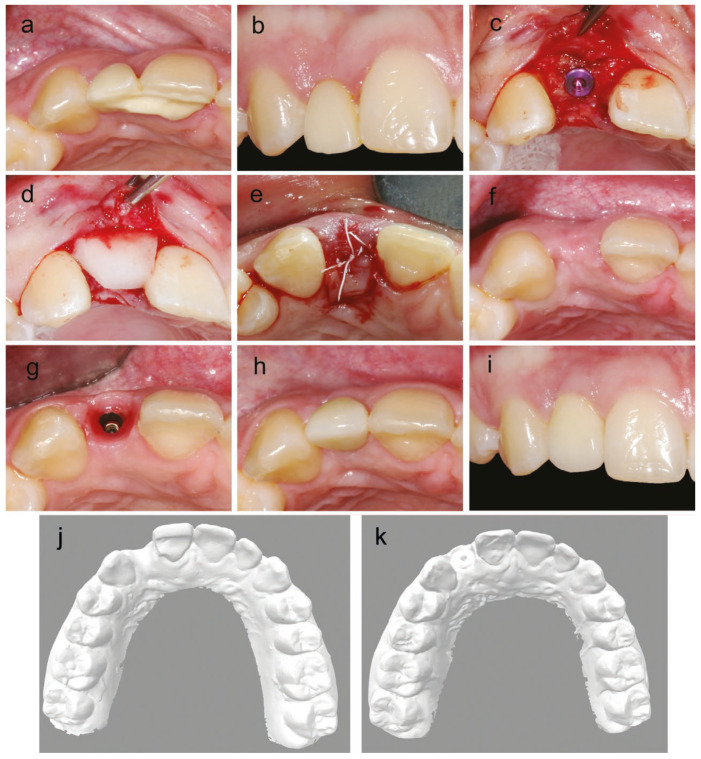
A clinical case illustrating soft-tissue augmentation with implant placement in a patient with a congenital absence of the upper right lateral incisor. (**a**) Occlusal view before treatment. (**b**) Buccal view before treatment. (**c**) Full thickness flap on top of the ridge with implant placed and split thickness flap on buccal side. (**d**) Collagen matrix placed underneath the buccal split flap and on top of the dental implant. (**e**) Primary wound closure with a palatal island flap to reduce the compression force of the buccal flap on the matrix. (**f**) Clinical situation (occlusal view) after 82 days just prior to re-entry. (**g**) Occlusal view of the emergence profile after soft tissue healing. (**h**) Occlusal view of the final prosthetic. (**i**) Buccal view of the final crown. Bottom panels show .stl files of the casts at (**j**) the pre-treatment and (**k**) post-treatment.

Soft-tissue health and implant survival and success were assessed at final prosthesis delivery and at the last follow-up. The Jemt’s papilla score distribution had improved significantly from final prosthesis delivery to the last follow-up (*p* = 0.005), with the optimal score of 3 at 35 sites and the score of 2 at 15 sites at final prosthesis delivery, while at the last follow-up, almost all sites (n = 46) received the score of 3 and only four were scored as 2. The keratinized tissue height remained stable, with a mean and standard deviation (SD) of 3.1 ± 1.0 mm at prosthetic delivery and 3.1 ± 1.0 mm at the last follow-up. BOP and plaque accumulation were evaluated at the last follow-up and confirmed the overall good health of the peri-implant soft tissue, with most sites (n = 39; 78%) showing no bleeding on probing, the mean generalized BOP of 14.4 ± 14.0, and the mean approximal plaque index (API) of 27.6 ± 16.4. All implants have survived and have been rated as successful throughout the study period, yielding the implant survival and success rates of 100%. A sample clinical case is shown in Figure 3.

#### 3.2.3. Patient Satisfaction

Implant-level patient ratings of their satisfaction with the esthetics of the restoration were good for 41 (82%) and acceptable for 9 (18%) final restorations; no poor esthetic rating was reported.

#### 3.2.4. Volumetric Analysis

**Figure 4:**
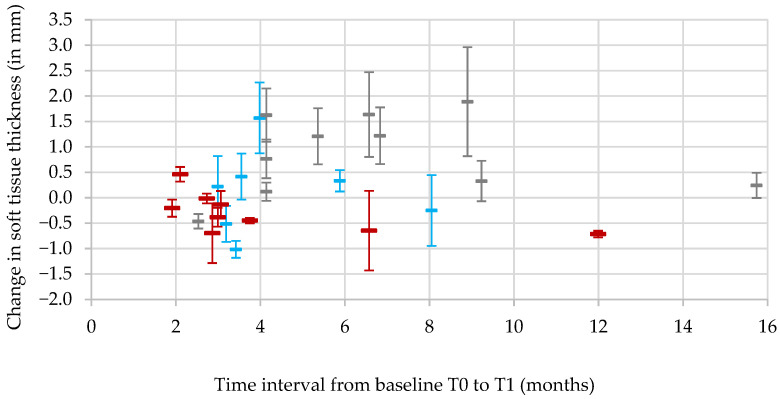
Soft-tissue thickness changes in relation to time interval from T0 to T1. Each augmentation site was measured at the same four positions at pre-treatment (T0) and at the time of impression-taking for the final prosthesis (T1). Measurements associated with immediate placement are shown in red, with early placement (5–8 weeks post-extraction) in blue, and with late placement (more than 8 weeks post-extraction) in gray. Positive values indicate soft-tissue gain and negative values indicate soft-tissue loss. Markers represent the mean and error bars standard deviation.

Plaster casts taken at pre-treatment (T0) and at the time of impression-taking for the final prosthesis (T1) were available for 26 soft-tissue-augmented sites from 23 patients to analyze volumetric changes in the soft tissue contour. The mean time from pre-treatment and final cast was 5.3 ± 3.3 months (range: 1.9–15.7 months). Overall, the change in soft-tissue thickness was minimal, with the mean of the individual linear measurements performed on the vestibular region of the augmented area of 0.25 ± 0.82 mm (range: −1.02 to 1.89 mm). This change was independent of time interval between T0 and T1 (Spearman’s correlation coefficient of 0.289; Pearson’s correlation coefficient of 0.142), indicating stability of the soft-tissue volume around the augmented sites (Figure 4). The null hypothesis of no correlation was not rejected (*p* = 0.1514 and *p* = 0.4902, respectively)**.**

To evaluate the possible influence of the placement protocol (fresh extraction sockets vs. early, i.e., 5–8 weeks post-extraction vs. late, i.e., more than 8 weeks post-extraction) on the STA outcome, the overall mean of the individual linear measurements performed on the vestibular region of the augmented area was calculated by implant placement protocol and compared using descriptive statistics. The immediate placement group, i.e., placement into fresh extraction sockets, showed an overall loss in mean tissue thickness of −0.31 ± 0.38 mm. By contrast, both the early and the late implant placement group showed an overall mean tissue gain of 0.11 ± 0.82 mm and 0.86 ± 0.78 mm, respectively (Figure 4). No statistically significant difference between variance in the three groups was detected (with *p*-values for fresh socket immediate placement, healed early placement, and healed delayed placement being 0.051, 0.0582, and 0.846, respectively).

### 3.3. Immunohistochemical Analysis

**Figure 5:**
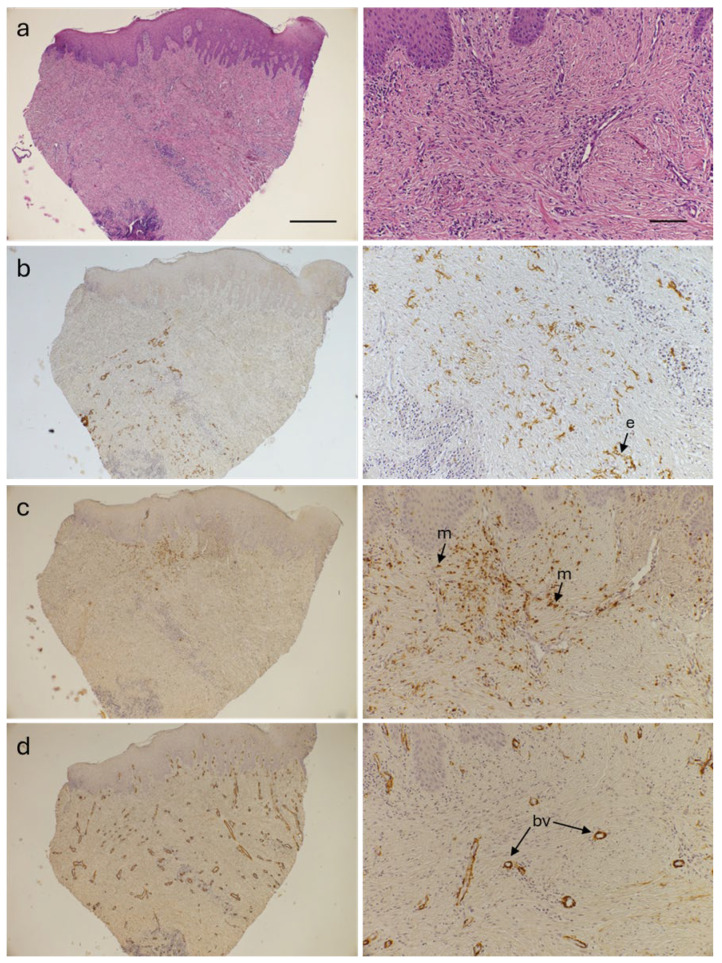
Immunohistochemical analysis of biopsy sample from a patient who underwent a late implant placement. The tissue was collected coronally to the implant 62 days after placement with simultaneous STA. Left panels, low magnification micrograph of an entire section: right panels, high magnification of the same micrograph. Scale bars: 500 μm (top left panel) and 100 μm (top right panel). (**a**) Hematoxylin and eosin staining to visualize overall tissue organization. (**b**) Elastin ‘e’ staining to visualize CM remnants (arrows pointing to elastin) in the submucosal layer. Note the resorption of the material (overall lack of discernible structure). (**c**) CD68 staining to visualize macrophages ‘m’. (**d**) α-SMA (smooth muscle cells) staining to visualize blood vessels ‘bv’. Note the vascularization throughout the section.

The punch re-entry procedure administered coronally to the implant sites (not involving any additional steps beyond abutment placement) provided material for biopsies which were collected (Figure 5a) for only those patients (n = 3) whose keratinized mucosa was sufficiently abundant for this approach (applicable to a total of five augmented sites, with one patient qualifying at three sites). The mean submerged healing time was 84.0 ± 19.1 days, allowing us to evaluate matrix integration. The remnants of the CM (as denoted by elastin stain) appeared in the submucosal layer in the biopsy taken after 62 days (Figure 5b) and the one harvested after 94 days of healing. In the lamina propria, low numbers of macrophages were present in the samples collected at the later points in one at 94 and three at 96 days post-procedurally, while moderate levels were visible in the biopsy taken after 62 days (Figure 5c), which is expected considering the short healing time in this case. Only one case of a CD68 negative foreign-body giant cell was found. In all cases, many blood vessels and extensive microvasculature were found underneath the epidermis (Figure 5d). These findings indicate healthy vascularization and minimal inflammation of the soft tissue augmented with CM (Figure 5).

## 4. Discussion

The present study evaluated the clinical performance of a non-crosslinked porcine collagen matrix with uniquely oriented open pore structure for STA. It showed a low rate of post-surgical complications (8% at 1-week and 4% at 3-week follow-up) with the majority of patients (66%) reporting minimal pain (1 out of 10), and no patient experiencing pain above 5. The keratinized mucosa remained stable from prosthetic delivery to the last follow-up (3.1 ± 1 mm), and approximately three quarters of the sites had no bleeding on probing or presence of plaque. Of 50 sites, 92% displayed optimal Jemt’s papilla at the last follow-up. The volumetric analysis indicated stability, and 100% of implants were deemed surviving and successful. Patients perceived esthetic outcomes as either good or acceptable.

As observed in studies with collagen membranes used for GBR procedures, chemical cross-linking can result in lower biocompatibility and tissue integration, higher immune reactions, and, therefore, increased complication rates such as incidence of wound dehiscence [38,39,40,41,42,43]. Similar findings were demonstrated for collagen matrices for STA procedures in animal models [24]. In the present study, the use of CM, which was not chemically cross-linked, resulted in a low rate of wound dehiscence of 2% (n = 1) and only one case of central matrix exposure (2%). Furthermore, immunohistochemical analysis revealed healthy matrix integration with good vascularization and no signs of inflammation such as material encapsulation or foreign-body reactions. Nonetheless, the remnants of the CM grafts were still visible, as evidenced by the presence of elastin in two of the three biopsies. This finding is consistent with the fact that elastin degradation in humans is considerably slower than in animal models [44] and suggests an inter-patient variability possibly associated with patient metabolism, defect size, etc. The remnant layer also showed presence of normal soft tissue along with blood vessels, indicating that the migration of host cells occurred with the resorption and remodeling of the collagen matrix. These results imply that the matrix serves as an efficient framework for populating living cells (e.g., fibroblasts), which is subsequently remodeled to natural soft tissue via a true regeneration process. Although a recent animal study revealed that the porous and elastic structure supported cellular migration and proliferation, it also made matrices susceptible to compression, which could delay tissue integration and elevate inflammatory response [45]. On a molecular level, the mechanical heterogeneity of the soft biomaterial comprising collagen fibers of varying thickness and length (as in case of CM used in the present study) may evoke a mode of self-stimulation of migrating cells and increase growth factor signaling that drives endogenous healing cascades [46]. In contrast to bulk hydrogels that exhibit uniform elastic modulus in all directions, the heterogeneous biomaterials can mimic the nature of a fracture hematoma and thereby promote enhanced healing [45,47]. These studies illuminate the importance of performing sufficient flap release avoiding exposed grafting material and over compression, as well as the need for further evaluation of heterogeneous collagen matrices.

Analysis of the tissue-thickness change showed that STA with CM, simultaneous with implant placement (either immediate or early), suggests the stability of soft-tissue dimensions (Figure 3). Since the highest resorption of the alveolar ridge width (about two-thirds) occurs in the first three months after tooth extraction, both immediate and early implant placement indications are impacted [1]. In these cases, the additional augmentation of soft tissue compensated for the volume loss. These results are consistent with those of another case series that used the same substitute material [48]. Similarly, other collagen matrices for soft-tissue thickening around dental implants have shown soft-tissue gain: 0.77 mm with simultaneous STA [49] or 1.1 mm for subsequent STA [50]. STA with autologous connective tissue grafts also showed a broad range of soft-tissue gain, with higher values that range between 0.7 mm and 1.43 mm [51]. As soft-tissue substitute materials tend to yield lower gains in tissue thickness, autologous CTGs remain the gold standard [17,52,53,54]. However, with the growing awareness of the importance of the patient’s experience, the minimally invasive nature of CM may outweigh the benefits of a more favorable endpoint of the autologous grafts.

As expected, the use of CM during surgery was simpler and less time-consuming compared with the harvesting of autologous soft-tissue grafts [55]. CM, whether dry or wet, was easy to shape using scissors or blades. The matrix could be stitched easily, and the suture-retention force was high enough to perform the envelope or tunneling techniques. The use of CM led to significant volume gain of the augmented area right after surgery (partly due to post-operative swelling), but resulted in significant tissue reduction during the healing phase, which is in agreement with other studies on soft-tissue substitutes [48,49].

Patient assessments in the present study showed low post-surgical discomfort and pain, and high levels of esthetic satisfaction at final prosthesis placement, which is consistent with the recent systematic reviews [18,19]. The presented treatment concept with simultaneous application of CM and implant placement, followed by prosthetic loading, led to 100% implant survival and success rates independent of the indication and loading protocol. Healthy and stable peri-implant soft-tissue dimensions were observed in patients at their last follow-up visit. These clinical parameters for soft-tissue health are comparable with those reported in a meta-analysis on the effect of STA on peri-implant health [10].

The main limitations of this study are the lack of a time course with the repeated volume measurements of the same sites at the later points to verify the stability of the gain, and the absence of a control group such as CTGs or other CMs materials to directly compare the outcomes of the creos^TM^ mucogain. Due to its retrospective design, this study was limited to the data routinely collected and available in the clinic’s electronic records, some of which were not quantitative (i.e., API). As this occurred as a part of daily practice, the tissue was collected during re-entry and preserved for histological assessment from only those sites, where sufficient mucosa for this treatment approach was available. While the small sample size is a limitation that may lead to sampling bias, the histological results demonstrate promising outcomes supported by results from other publications where the same material was successfully utilized as a nerve guide or facilitated dermal regeneration [22,56]. Similarly, although only about half of the plaster casts were readable/available for volumetric analysis after a period of time, it was caused by technical issues and not subject to selection bias. Although this heterogeneity can pose challenges for standardization, it provides a more authentic view of treatment performance and outcomes—insights that are critical for refining protocols, expanding indications, and improving predictability in routine care. To draw more definitive conclusions regarding soft-tissue stability and patient’s experience, a randomized clinical trial with a control group should include multiple direct tissue-thickness measurements over time to yield exact and more conclusive results, as well as the long-term patient-reported outcome measures, which could potentially reveal whether the seemingly greater gains from CTGs, as compared to CM-based grafts, compensate for the discomfort from having a second surgical site.

## 5. Conclusions

The collagen-based matrix soft-tissue substitute CM demonstrated high biocompatibility and predictable clinical performance for soft-tissue volume augmentation around dental implants. It resulted in good soft-tissue integration of the matrix and low complication rates. It also aided in stable soft-tissue conditions with low post-surgical discomfort, high esthetic satisfaction, and 100% implant survival.

## Figures and Tables

**Table 1:** Baseline patient and surgical characteristics.

Patient Characteristics		n (%)
Gender	Female	25 (55.6%)
	Male	20 (44.4%)
Age (mean ± SD)		41.4 ± 13.1 years
Smoking status	Non-smoking	35 (77.8%)
	Smoking, ≤10 per day	10 (22.2%)
Periodontitis	Yes	4 (8.9%)
	No	41 (91.1%)
Health history	Adiposities, high blood pressure	1 (2.2%)
	Blood thinner due to stroke ^1^	1 (2.2%)
	Diabetes type I	1 (2.2%)
Implants per patient	1	41 (91.9%)
	2	3 (6.7%)
	3	1 (2.2%)
Implant characteristics		n (%)
Implant type	NobelActive CC	31 (62%)
	NobelParallel CC	13 (26%)
	NobelReplace CC	3 (6%)
	NobelReplace Straight Groovy	2 (4%)
	NobelPearl	1 (2%)
Implant site/surgical characteristics		n (%)
Reason for tooth extraction	Caries	19 (38%)
	Caries and endodontic failure	1 (2%)
	Congenitally missing	2 (4%)
	Endodontic failure	12 (24%)
	Horizontal fracture trauma, loose tooth	3 (6%)
	Periodontal problems	1 (2%)
	Perio-endo problems	4 (8%)
	Vertical fracture	1 (2%)
	Not reported	7 (14%)
Location	Maxilla total	37 (74%)
	Maxilla anterior	19 (38%)
	Maxilla posterior	18 (36%)
	Mandible total	13 (26%)
	Mandible anterior	2 (4%)
	Mandible posterior	11 (22%)
Insertion torque (mean ± SD)		50.60 ± 17.10 Ncm
Site type/Implant placement protocol	Extraction socket (immediate)	19 (38%)
	Healed, 5–8 wk post-extraction (early)	14 (28%)
	Healed, >8 wk post-extraction (delayed)	17 (34%)
Surgical access	Flap	20 (40%)
	Flapless	30 (60%)
Bone grafting	Yes	28 (56%)
	GBR	8 (16%)
	Gap augmentation	14 (28%)
	Sinus elevation	6 (12%)
	No	22 (44%)
Bone-grafting material	NanoBone	1 (2%)
	Bio-Oss	17 (34%)
	creos xenogain	10 (20%)
STA technique	Envelope technique	22 (44%)
	Trapezoid, mucosal and mucoperiosteal flap	25 (50%)
	Coronally advanced flap	3 (6%)
Soft-tissue healing	Closed	23 (46%)
	Transmucosal	27 (54%)

^1^ This patient was also diagnosed with periodontitis.

## Data Availability

Data supporting the study findings are not openly available due to privacy concerns. De-identified data that support the findings of this study are obtainable from the authors upon reasonable request.

## Supplementary Materials

The following supporting information can be downloaded at: https://www.mdpi.com/article/10.3390/bioengineering12121324/s1, Figure S1: Staining Panels.

## Abbreviations

The following abbreviations are used in this manuscript:

BOP	Bleeding on probing
CM	Collagen matrix
CTG	Connective tissue graft
GBR	Guided bone regeneration
PFM	Porcelain-fused-to-metal
RECORD	Reporting of studies Conducted using Observational Routinely collected health Data
STA	Soft-tissue augmentation
XCM	Xenogeneic collagen matrix
