# Supplementary figures and images for: Soft-Tissue Volume Augmentation at Dental Implant Placement Using Collagen-Based Matrix Characterized by Oriented Open Pore Structure: A Retrospective Study with a Median Follow-Up of 17 Months
Source: Bioengineering (Basel). 2025 Dec 4;12(12):1324. doi: 10.3390/bioengineering12121324 (PMC12729357; doi:10.3390/bioengineering12121324)

| Staining | 50x                                                                                  | 100x                                                                                  | 200x                                                                                  |
|----------|--------------------------------------------------------------------------------------|---------------------------------------------------------------------------------------|---------------------------------------------------------------------------------------|
| Herovici | 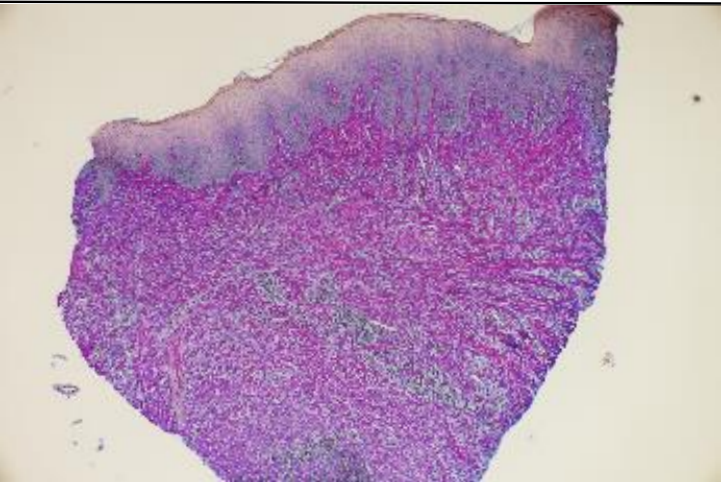   | 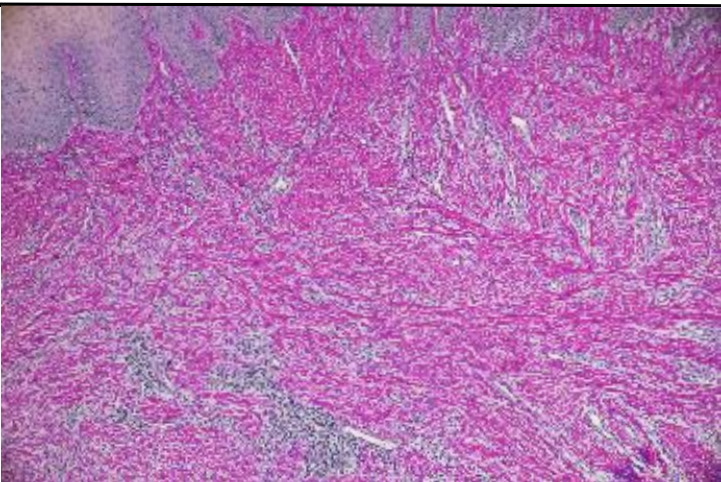   | 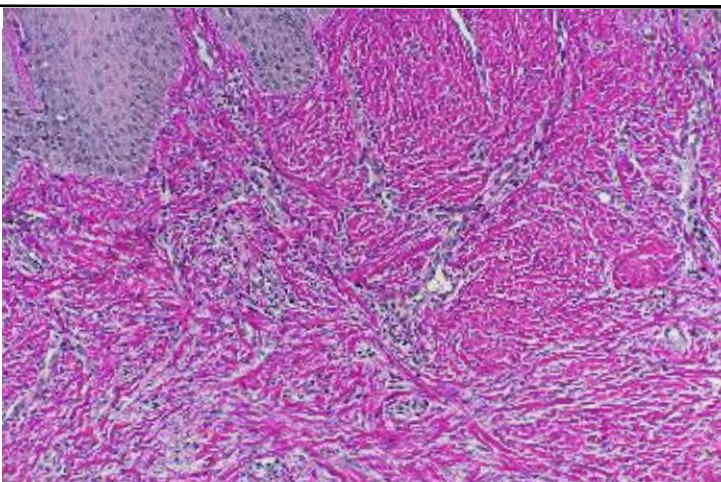   |
| vWF      | 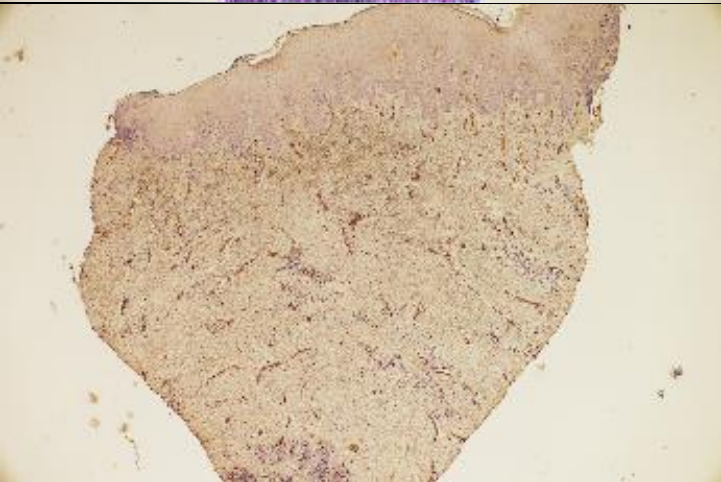  | 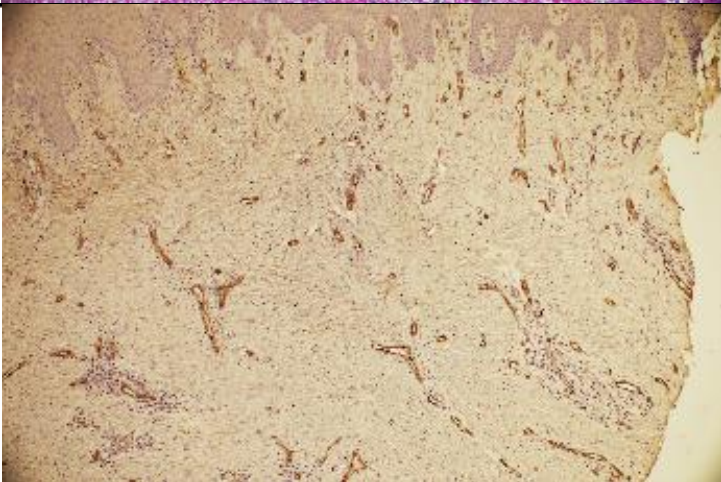  | 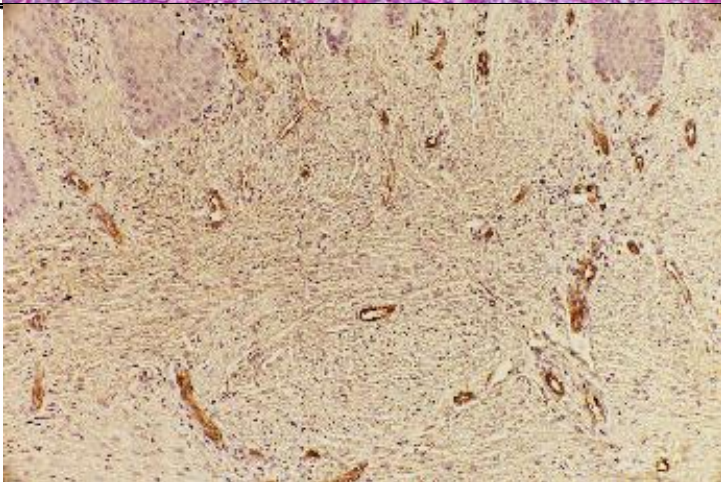  |
| CD34     | 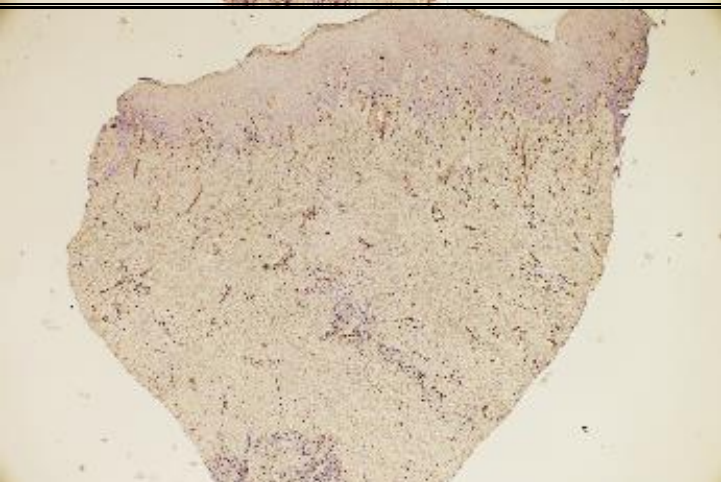 | 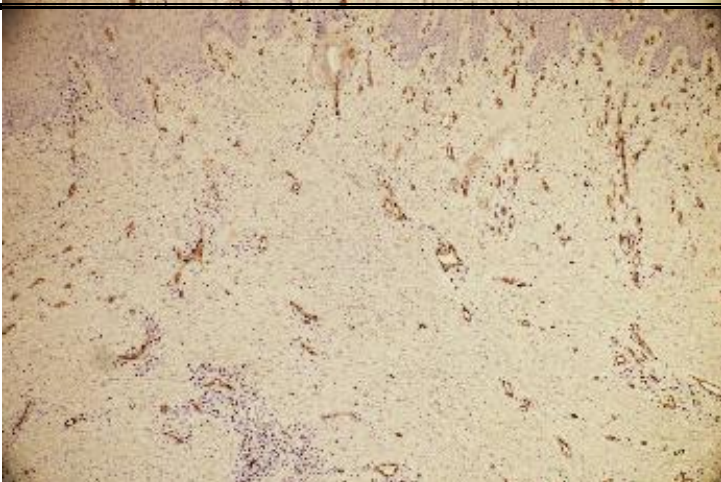 | 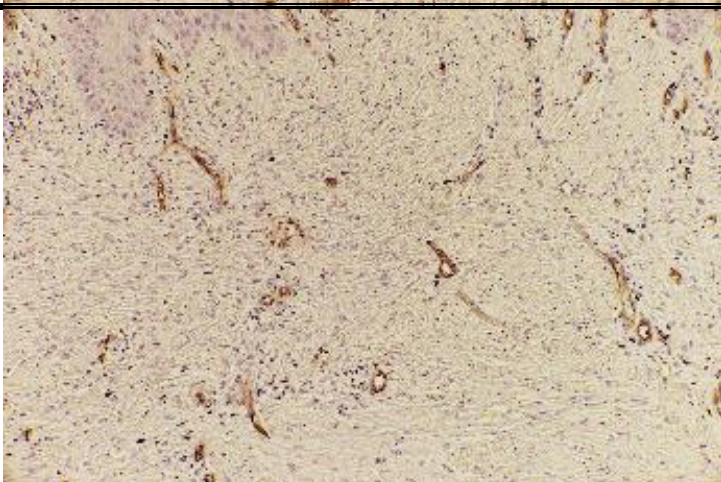 |
| collIV   | 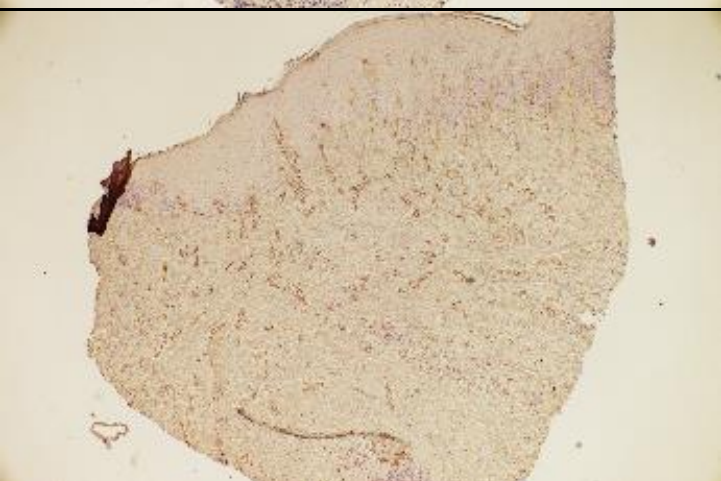 | 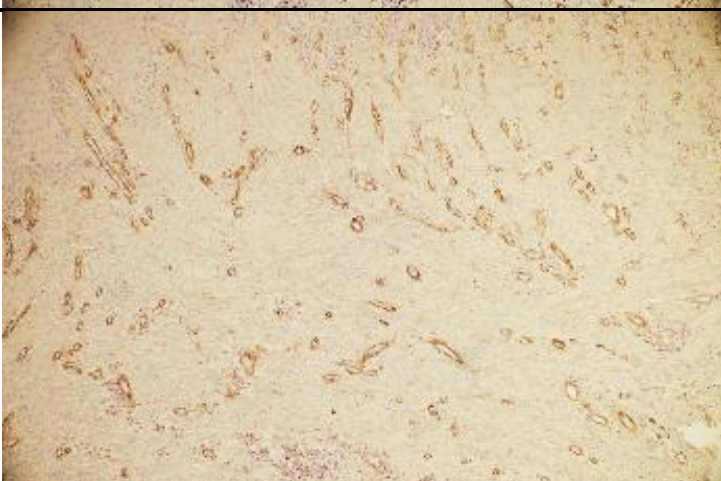 | 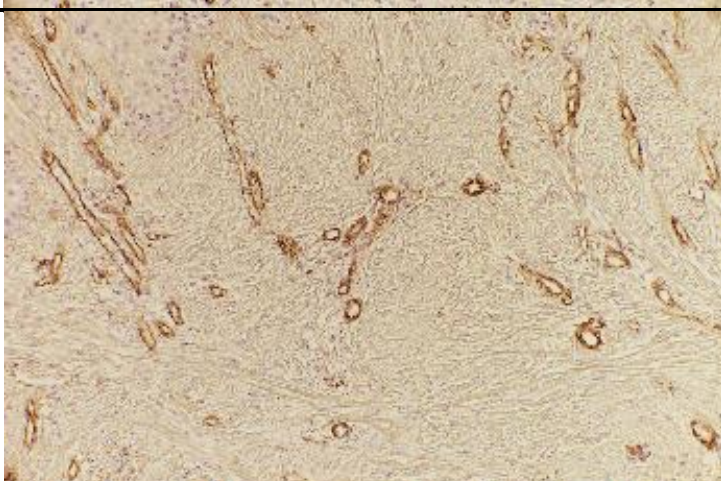 |

Supplement: Supplementary file 1 [file bioengineering-12-01324-s001.zip › Staining Panels.pdf]
